# Novel compound heterozygous variants in *EMC1* associated with global developmental delay: a lesson from a non-silent synonymous exonic mutation

**DOI:** 10.3389/fnmol.2023.1153156

**Published:** 2023-04-28

**Authors:** Ge Wang, Yanli Wang, Chao Gao, Wanqin Xie

**Affiliations:** ^1^Department of Neurology, Xiangya Hospital, Central South University, Changsha, China; ^2^Department of Rehabilitation Medicine, Children's Hospital Affiliated to Zhengzhou University, Henan Children's Hospital, Zhengzhou, China; ^3^National Health Committee Key Laboratory of Birth Defects for Research and Prevention, Hunan Provincial Maternal and Child Health Care Hospital, Changsha, China

**Keywords:** global developmental delay, endoplasmic reticulum-membrane protein complex, synonymous mutation, gene splicing, genetic diagnosis

## Abstract

**Background:**

The endoplasmic reticulum-membrane protein complex (EMC) as a molecular chaperone is required for the proper synthesis, folding and traffic of several transmembrane proteins. Variants in the subunit 1 of EMC (*EMC1*) have been implicated in neurodevelopmental disorders.

**Methods:**

Whole exome sequencing (WES) with Sanger sequencing validation was performed for a Chinese family, including the proband (a 4-year-old girl who displayed global developmental delay, severe hypotonia and visual impairment), her affected younger sister and her non-consanguineous parents. RT-PCR assay and Sanger sequencing were used to detect abnormal RNA splicing.

**Results:**

Novel compound heterozygous variants in *EMC1*, including the maternally inherited chr1: 19566812_1956800delinsATTCTACTT[hg19];NM_015047.3:c.765_777delins ATTCTACTT;p.(Leu256fsTer10) and the paternally inherited chr1:19549890G> A[hg19];NM_015047.3:c.2376G>A;p.(Val792=) are identified in the proband and her affected sister. RT-PCR assay followed by Sanger sequencing reveals that the c.2376G>A variant leads to aberrant splicing, with retention of intron 19 (561bp) in the mature mRNA, which is presumed to introduce a premature translational termination codon (p.(Val792fsTer31)).

**Conclusion:**

Novel compound heterozygous variants in *EMC1* have been identified in individuals with global developmental delay. Non-silent synonymous mutations should be kept in mind in genetic analysis.

## Introduction

Global development delay (GDD) depicts a clinically and etiologically heterogeneous group of neurodevelopmental disorders as evidenced by significant delays in two or more developmental domains in patients under 5 years old. The term “intellectual disability” (ID) is applied to patients with GDD who are ≥5 years old (Abu-Safieh et al., 2013). By taking advantage of next-generation sequencing techniques, e.g., copy number variations sequencing (CNV-seq) and whole exome sequencing (WES), significantly increased Mendelian causes for GDD/ID have been identified, including a growing number of micro-deletions/duplications and monogenic genes (Bartoszewski et al., 2016; Broly et al., 2022; Bryen et al., 2023).

The highly conserved endoplasmic reticulum membrane protein complex (EMC), consisting of 10 subunits (EMC1-10), acts as a molecular chaperone for the proper synthesis, folding and traffic of several transmembrane proteins, such as rhodopsin, nicotinic acetylcholine receptor, and transmembrane receptor of Wnt signaling (Cabet et al., 2020; Chung et al., 2022). The *EMC1* gene (OMIM ^*^616846), located on 1p36.13, encodes a full-length protein of 993 amino acid residues that is ubiquitously expressed in tissues and organs. Depletion of EMC1 is related to retinal degeneration, neural crest development defect, and glial abnormality in experimental animal models (Cabet et al., 2020; Chung et al., 2022; Dhindsa et al., 2022). To date, about a dozen of *EMC1* variants, including missense, splice site, non-sense, and frameshift variants have been identified and associated with highly variable clinical presentation, ranging from isolated retinitis pigmentosa or congenital heart disease to severe global development delay (Harel et al., 2016; Geetha et al., 2018; Gao et al., 2019; Jin et al., 2021; Dhindsa et al., 2022).

Synonymous mutations that alter the sequence of a gene but not the sequence of the encoded protein are usually referred to as “silent” (Lemire et al., 2021). Notably, pathogenic synonynous mutations have been characterized in a diversity of genetic disorders, including the neurological diseases amyotrophic lateral sclerosis (ALS) (Marquez et al., 2020), autism spectrum disorders (ASDs) and schizophrenia (SCZ) (McCarthy et al., 2017). Mechanistically, the reported pathogenic synonymous mutations have effects by altering mRNA splicing, structure, stability or translation efficacy to affect protein function or expression level (Moeschler and Shevell, 2014). Non-silent synonymous mutations are uncommon, and thus they are easily overlooked in genetic analysis, leading to non-conclusive results in genetic diagnosis (Satoh et al., 2015; Postel et al., 2022).

In this study, we investigated the genetic etiology of a Chinese family affected by global developmental delay.

## Materials and methods

### Participants and study approval

The study included the proband (a 4-year-old girl), her affected younger sister (15 months old) and her non-consanguineous parents of a Chinese family. The family was previously provided with a non-conclusive result in genetic testing. Written informed consent for participation in the study and for publication was obtained. The study was approved by the Ethics Committee of the Xiangya Hospital of Central South University.

### Whole exome sequencing and data analysis

Trio whole-exome sequencing (WES) was performed for the proband and her unaffected parents to identify the causative gene. Genomic DNA was isolated from venous blood sample with Qiagen DNA Blood Mini kit (QIAGEN, GmbH, Germany). The purified DNA was subjected to fragmentation using sonication (cat: FB705, Thermo Fisher, Waltham, MA, United States), followed by hybrid capture with xGen Exome Research Panel v2.0 (Integrated DNA Technologies, Inc., Coralville, IA, United States), enrichment, and sequencing on the DNBSEQ-T7 platform. The clean data were aligned to the Genome Reference Consortium Human genome build 37 (GRCh37) using Burrows-Wheeler Aligner (BWA) software, and variant calling was performed using Genome Analysis Toolkit (GATK). The ANNOVAR tool was used for variant annotation, and standard filtering was conducted (Stephenson et al., 2022). In this study, we retained all synonymous variants. MaxEntScan (https://www.genes.mit.edu/burgelab/maxent/Xmaxentscan_scoreseq_html) and dbscSNV (http://www.liulab.science/dbscsnv.html) were used to predict the influence on splicing. Pathogenicity was interpreted according to the American College of Medical Genetics (ACMG) guidelines. Sanger sequencing was used to further validate the candidate variants in all family members.

### RT-PCR and cDNA sequencing

The total RNA extraction kit (Tiangen Biotech Co. Ltd, Beijing, China) was used to extract RNA from the venous blood samples of the proband and healthy control. Reverse transcription was carried out with the superscript IV first-strand synthesis system (Thermo Scientific, Shanghai, China). Primer sets for cDNA amplification were designed using Oligo 7. The forward primer (5′-TACAGCGGATCGTCAAGGTG-3′) and reverse primer (5′-AACAAGTGGACTCCAGACC C-3′) were mapped to 1 or 2 exons upstream/downstream of the mutation site. The amplification reaction was performed using KAPA2G Robust HotStart PCR Kit (Roche) on Hema-9600 equipment. The PCR products were analyzed by 3.0% agarose gel electrophoresis and Sanger sequencing.

## Results

### Clinical presentation

The proband, a 4-year-old girl, is the first child born to non-consanguineous Chinese parents. She showed delayed developmental milestones. At 8 months, she had manifestations of language and motor developmental delay, neck weakness, poor palmar grasp, and hypotonia. On examination at 4 years, we observed that she could only pronounce the simple sounds “bà” and “mā” of Mandarin Chinese bàba and māma, and had difficulty in independent sitting, standing, and walking. In addition, horizontal nystagmus and retinal dystrophy were recognized. The head circumference of patient history and physical examination was in the normal range. Brain MRI showed normal cerebral and cerebellar volume and structure. Longitudinal MRI did not show progressive cerebellar atrophy. There was no history and electroencephalogram suggestive of seizures. At the age of 8 months, the proband received genetic testing (WES and SNP-array tests) at a local hospital, but no conclusive information was provided. The younger sister of the proband presented with severe hypotonia during the neonatal period. The head circumference and brain MRI of the younger sister were normal. In light of two affected children in the family, the couple was referred to our institution for genetic counseling.

### Novel variants in the EMC1 gene

**Figure 1:**
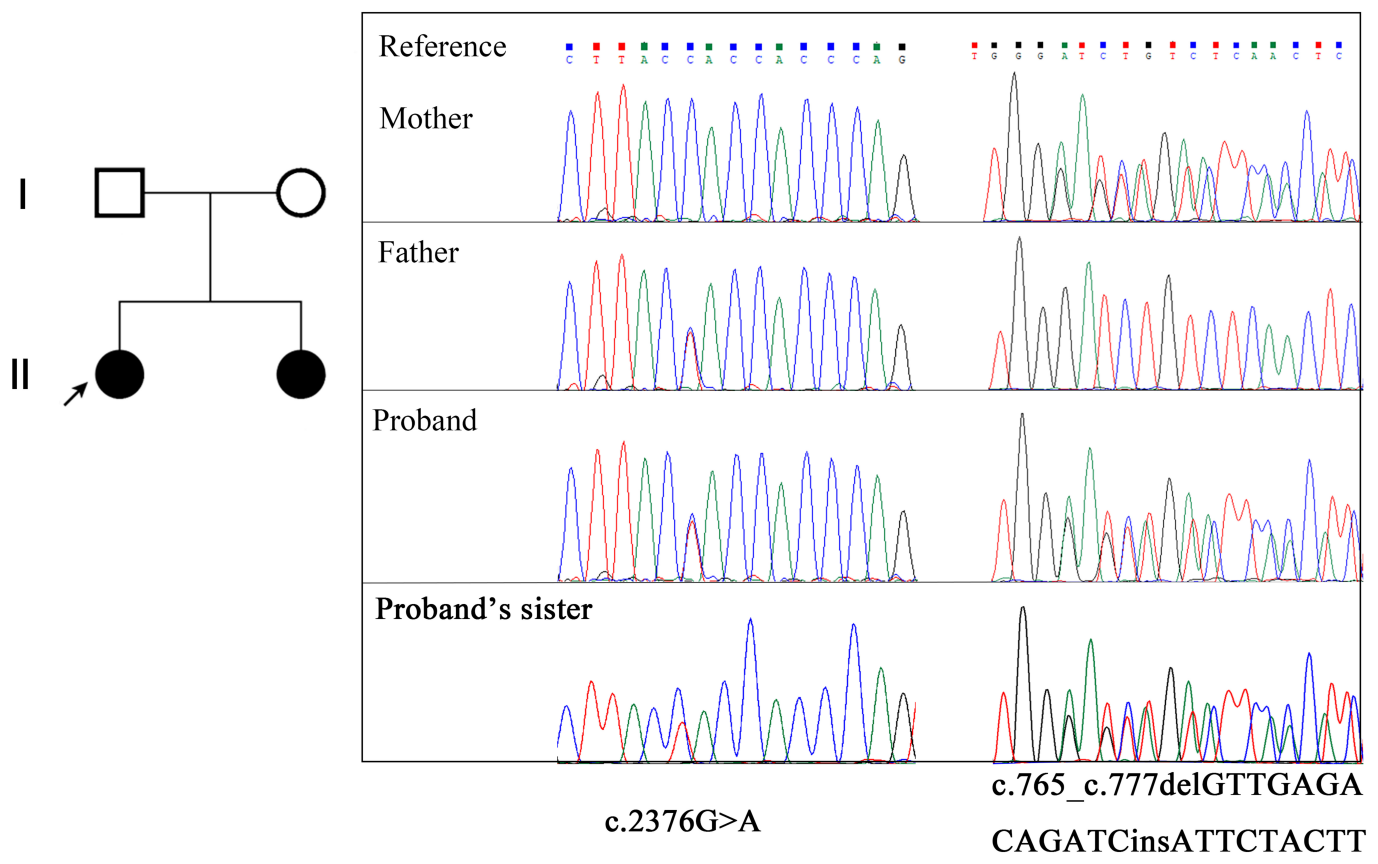
Pedigree of the family and *EMC1* variants confirmed by Sanger sequencing. The proband is indicated with an arrow.

**Figure 2:**
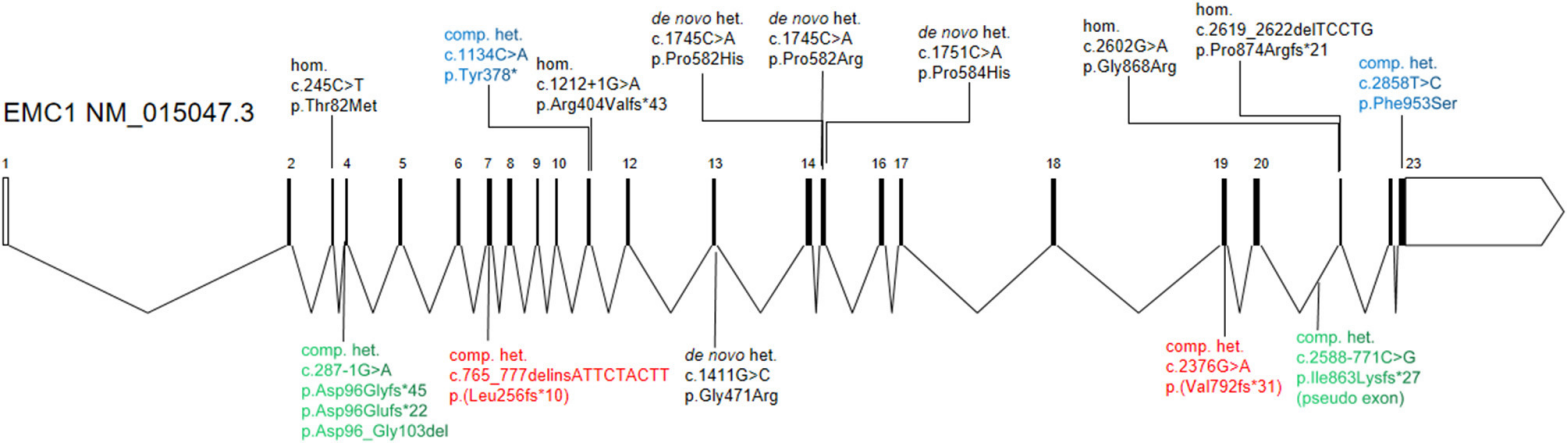
Schematic diagram of the *EMC1* gene structure and variations associated with global developmental delay. The compound heterozygous variants from current study (red color) and two previous studies (green and blue colors) are highlighted. The previously reported homozygous or heterozygous variants are shown in black.

WES followed by Sanger sequencing validation identified compound heterozygous mutations of *EMC1* (NM_015047.3) in the proband, comprising the maternally inherited c.765_c.777delinsATTCTACTT mutation in exon 7 and the paternally inherited c.2376G>A mutation in exon 19 (Figures 1, 2). Sanger sequencing confirmed that the affected younger sister also carries the compound heterozygous mutations. The c.765_c.777delinsATTCTACTT variant is predicted to cause a frameshift, resulting in an early truncated protein p.(Leu256fsTer10). The synonymous variant c.2376G>A;p.(Val792=) is predicted to affect splicing according to the MaxEntScan (8.15->4.16) and dbscSNV (0.9998|0.9900) softwares. Both variants have not been indexed in ExAC, gnomAD, and dbSNP databases. According to the ACMG guidelines, the c.765_c.777delinsATTCTACTT variant and the c.2376G>A variant were classified as “likely pathogenic” (PVS1 + PM2) and “uncertain significance” (PM2 + PM3 + PP3), respectively.

### Aberrant splicing of the EMC1 c.2376G>A variant

**Figure 3:**
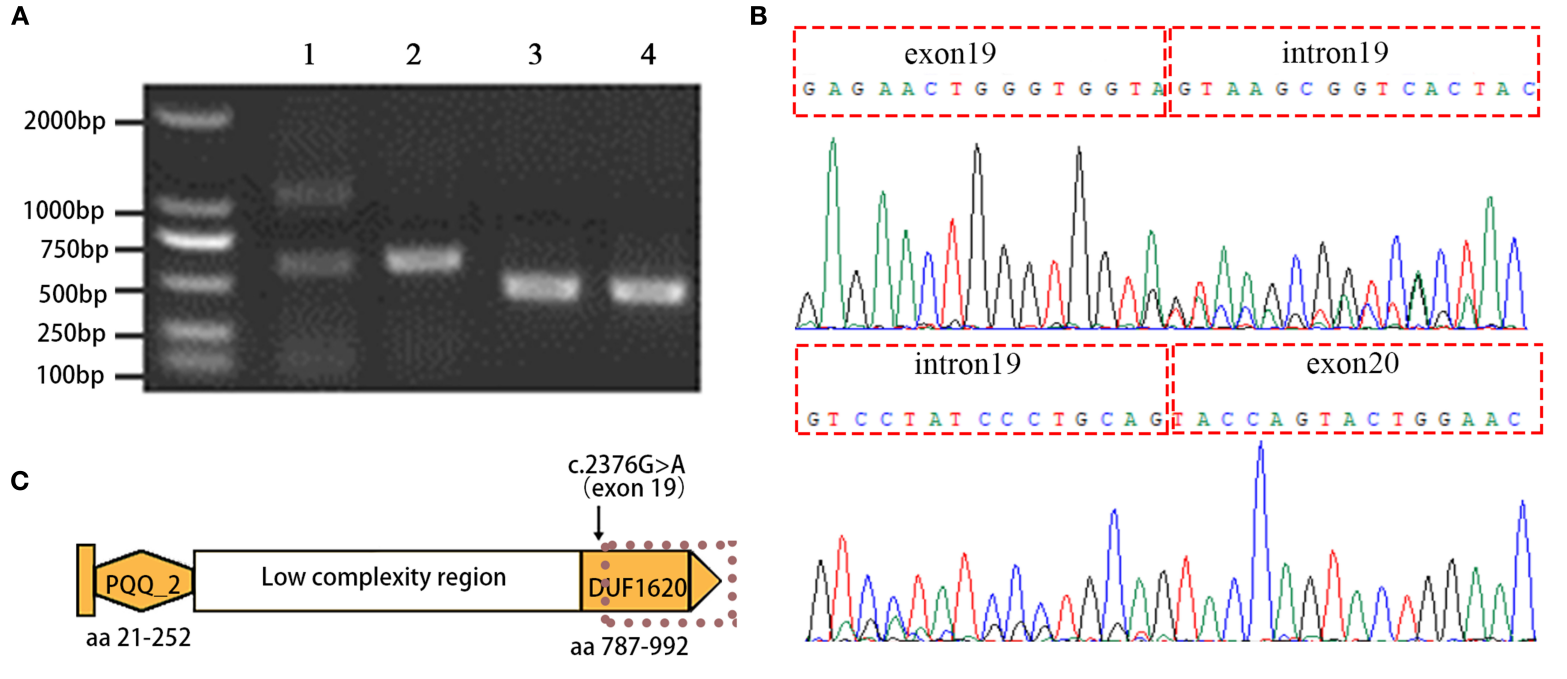
Aberrant splicing of the *EMC1* c.2376G>A variant is detected by using RT-PCR and Sanger sequencing. **(A)** Agarose gel electrophoresis image shows the RT-PCR products amplified by the primers spanning *EMC1* exons 18-22 with inclusion of the c.2376G>A mutation site in exon 19 (lane 1, the proband; lane 2 healthy control), and the primers targeting the upstream and downstream sequences of the c.765_c.777delinsATTCTACTT mutation (lane 3, the proband; lane 4 healthy control). **(B)** Sanger sequencing reveals the aberrant retention of intron 19 between exon19 and exon 20 in *EMC1* mRNA. **(C)** Schematic representation of the domains of EMC1. PQQ_2 represents the quinoprotein alcohol dehydrogenase-like domain and DUF1620 represents the uncharacterized domain of unknown function 1620. The dotted line indicates premature truncation of the protein due to the aberrant mRNA resulting from the c.2376G>A variant.

To detect the potential aberrant splicing of the c.2376G>A variant, specific primers were used to amplify the sequence spanning exon 18 through exon 22 with the synthesized cDNA templates. The wild-type allele of *EMC1* is expected to produce an amplicon of 690 base pairs according to the primers. Indeed, RT-PCR products as resolved by agarose gel electrophoresis showed that the healthy control had a single amplicon with expected size, whereas the proband had two amplicons, suggesting an aberrant transcript in addition to the normal-sized transcript (Figure 3A). The aberrant transcript was also detected in the paternal sample since the father carries the c.2376G>A variant, but not in the maternal and unrelated healthy control samples (Supplementary Figure 1). Sanger sequencing further revealed that the aberrant transcript results from full retention of intron 19 (561 bp) in the mature mRNA (Figure 3B; Supplementary Figure 1). Translation of the resultant mRNA is predicted to have the insertion of 31 non-coding amino acid residues following exon 19 and premature truncation of the protein due to a stop codon (p.(Val792fsTer31)), resulting in a loss of the C-terminal DUF1620 domain (amino acid residues 787-992) (Figure 3C). For comparison, an amplicon covering the mutation site c.765_c.777delinsATTCTACTT showed no apparent difference in the size of the RT-PCR products between the proband and the healthy control (Figure 3A). BLAST (https://blast.ncbi.nlm.nih.gov/Blast.cgi) analysis revealed that the transcript isoforms of *EMC1* are highly identical to each other and only minor in-frame gaps exist between the longest isoform 1 (NM_015047.3) and other isoforms (transcript variants 2-6). Therefore, the c.2376G>A and c.765_c.777delinsATTCTACTT variants are thought to affect all the known transcript isoforms. Based on the finding of altered splicing, the c.2376G>A variant was regraded as “likely pathogenic” (PS3+PM2) in terms of the ACMG guidelines.

## Discussion

EMC1 is a core subunit for the assembly and function of the EMC complex. Mutations in *EMC1* have been associated with GDD in several studies (Harel et al., 2016; Geetha et al., 2018; Gao et al., 2019; Sun et al., 2019; Jin et al., 2021; Dhindsa et al., 2022) (Figure 2), where the affected individuals showed variable clinical presentation with major findings such as global developmental delay, cerebellar atrophy, psychomotor retardation, hypotonia, and visual impairment (Supplementary Table 1). The clinical features of the proband in current report are highly similar to the phenotypes described in previous individuals, supporting a diagnosis of EMC1-related disorders. However, no progressive cerebellar atrophy was noticed in the proband at the age of 4 years. Intriguingly, the individuals who carry compound heterozygous variants in *EMC1* seem to less frequently present with symptoms such as scoliosis, postnatal microcephaly, dysmophic features and cerebellar atrophy than the individuals who carry heterozygous or homozygous variants in *EMC1* (Supplementary Table 1).

Exonic synonymous mutations are usually considered “silent” and as well as non-pathogenic. Although synonymous mutations are often detected by WES, insufficient attention has been paid (Satoh et al., 2015; Postel et al., 2022). Notably, synonymous mutations may have an impact on gene expression when they alter certain exonic motifs. For example, synonymous mutations adjacent to splice sites may alter gene splicing and have a functional consequence (Supek et al., 2014). In this study, the initial genetic testing for the proband did not reach a conclusive result. In our analysis, we employed trio-WES to explore the genetic cause, and the association between the clinical phenotype and the bi-allelic mutation of *EMC1* in the proband was implied. However, the consistency of genotype and phenotype was challenged by the undetermined pathogenicity of the synonymous mutation c.2376G>A (p.Val792=). Using RT-PCR and Sanger sequencing, we have proven that the synonymous mutation c.2376G>A leads to abnormal splicing, generating an aberrant mRNA with retention of intron 19, which is expected to produce an early truncated protein p.(Val792fsTer31). Our experimental evidence suggests that the *EMC1* c.2376G>A variant is most likely to be loss-of-function, and that bi-allelic loss of function in *EMC1* are the genetic cause for global developmental delay in the proband. In our study, we used RT-PCR combined with Sanger sequencing to detect the aberrant splicing of the *EMC1* exonic variant. In the case of intronic variants and UTR variants, RNA sequencing is an ideal tool to detect gene-specific altered splicing and expression (Takata et al., 2016).

Our experience shows that certain “silent” mutations, especially those detected in trans to loss-of-function mutations and related to autosomal recessive diseases, should not be overlooked during variant filtering and interpretation in WES. Further functional assays should be performed regarding these “silent” mutations. Overlooking these “silent” mutations might cause an unclear molecular diagnosis, which will increase challenges in the next pregnancy and also place a significant psychological and emotional burden on the family.

In summary, we have identified novel compound heterozygous variants in *EMC1* as the genetic cause in the individuals from a Chinese family affected with global developmental delay. To the best of our knowledge, this is the first report regarding pathogenic synonymous mutation in *EMC1*. Our findings suggest a more cautious attitude toward synonymous mutations in genetic analysis.

## Data availability statement

The data presented in the study are deposited in the Sequence Read Archive (SRA) repository, accession number PRJNA934148 (https://www.ncbi.nlm.nih.gov/sra/PRJNA934148).

## Ethics statement

The studies involving human participants were reviewed and approved by Ethics Committee of the Xiangya Hospital of Central South University. Written informed consent to participate in this study was provided by the participants' legal guardian/next of kin. Written informed consent was obtained from the individual(s), and minor(s)' legal guardian/next of kin, for the publication of any potentially identifiable images or data included in this article.

## Author contributions

GW, YW, and CG recruited the study participants and collected the clinical data. GW and YW performed the sequencing analysis and interpreted the data. CG and WX designed the study and reviewed all the data. GW and WX wrote the manuscript draft. All authors read and approved the final manuscript.
