# Supplementary material for: Novel compound heterozygous variants in EMC1 associated with global developmental delay: a lesson from a non-silent synonymous exonic mutation
Source: Front Mol Neurosci. 2023 Apr 28;16:1153156. doi: 10.3389/fnmol.2023.1153156 (PMC10175691; doi:10.3389/fnmol.2023.1153156)
Supplement: Supplementary file 1 [file Table_1.docx]

Supplemental Table 1 Variants in *EMC1* identified in individuals with global developmental delay

| Source | Current report | Bryen at al., 2023: | Chuang et al., 2022: Family A | Chuang et al., 2022: FamilyB | Chuang et al., 2022: Family C | Cabet et al., 2020 | Geetha et al., 2018 | Harel et al.,2016: Family 1 | Harel et al.,2016: Family 2 | Harel et al.,2016: Family 2 | Harel et al.,2016: Family 2 | Harel et al.,2016: Family 3 | Harel et al.,2016: Family 3 | Harel et al.,2016: Family 4 |
| --- | --- | --- | --- | --- | --- | --- | --- | --- | --- | --- | --- | --- | --- | --- |
| Individual |  |  |  |  |  |  |  | A3 | BAB3445 | BAB3446 | BAB4742 | BAB6896 | BAB6897 | BH4387_1 |
| Variants in EMC1 (NM_015047.3) | c.765_c.777delGTTGAGACAGATCinsATTCTACTT;p.Leu256fs*10);  c.2376G>A;p.Val792fs*31 (comp het) | c.287-1G>A; p.Asp96Glyfs*45, p.Asp96Glufs*22, p.Asp96Valfs*14; c.2588-771C>G; p.Ile863Lysfs*27 (comp het) | c.1745C>A; p.Pro582His (het) | c.1745C>G; p.Pro582Arg (het, mosaic) | c.1751C>A; p.Pro584His (het) | c.1134C>A; p.Tyr378Ter; c.2858T>C; p.Phe953Ser (comp het) | c.1212+1G>A;p.Arg404Valfs*43 (hom) | c.2619_2622delTCCT; p.Pro874Argfs*21 (hom) | c.245C>T; p.Thr82Met (hom) | c.245C>T; p.Thr82Met (hom) | c.245C>T; p.Thr82Met (hom) | c.2602G>A; p.Gly868Arg (hom) | c.2602G>A; p.Gly868Arg (hom) | c.1411G>C; p.Gly471Arg (het) |
| Age at last exam | 4 yr | 7 yr (deceased) | 4 yr | 15 yr | 5 yr | 10 yr | 4yr &10 mo | 4 yr | 13 yr | 5 yr | 3 yr | 10 yr | 12 yr | 12 yr |
| Global developmental delay | yes | yes (5 mo) | yes, profound | yes, profound | yes, profound |  | yes, severe | yes | yes | yes | yes | yes | yes | yes |
| Speech delay | two words | five spoken words | no speech | no speech | no speech | no speech | no speech | yes | yes | yes | yes | yes | yes | yes |
| Seizures | no | no | early onset,  refractory,  absences,  tonic, clonic and tonic-clonic  seizures,  myoclonia | absence | yes | no | early infantile, myoclonic jerks and generalized tonic-clonic | yes (subclinical) | no | no | no | no | no | no |
| Scolios | no | no | yes | yes | no | no | yes | yes | yes | yes | yes | no | no | yes |
| Postnatal microcephaly | no | 50th centile, brachycephaly and mid-temporal narrowing | no (-0.76SD); yet exhibited a postnatal decline in percentiles | yes | no | no | yes | 3rd-10th percentile (2yr9mo) | yes | yes | yes | no | no | 6^th^ percentile (18 mo) |
| Dysmorphic features | no | no | scaphocephaly | no | no | no | scaphocephaly, deep-set eyes | short upper lip, mild hypertelorism, retrognathia | deep-set eyes, gingival hyperplasia, short philtrum, retrognathia, persistent fetal fingerpads | deep-set eyes, gingival hyperplasia, short philtrum, retrognathia, persistent fetal fingerpads | deep-set eyes, gingival hyperplasia, short philtrum, retrognathia, persistent fetal fingerpads | deep-set eyes, retrognathia | deep-set eyes | low anterior hairline, dysplastic ears, gingival hyperplasia, micrognathia |
| Truncal hypotonia | yes | yes | yes | yes | yes | yes | yes | yes | yes | yes | yes | yes | no | yes |
| Increased tone in extremities |  |  | yes | yes | no | no |  | yes | yes | yes | yes | no | no | no |
| Diminished DTRs | no | absent deep tendon reflexes | yes | yes | yes in upper extremities, brisk reflexes in lower extremitie | no | yes | yes | yes | yes | yes | NR | no | yes |
| Dystonic posturing |  |  | yes | yes | no | no | yes | yes | yes | yes | yes | no | no | no |
| Ophthalmological abnormalities | horizontal nystagmus and retinal dystrophy | cortical visual impairment; bilateral cataracts at 6 yr (possibly due to self injurious behavior); intermittent esotropia | cortical visual impairment; no response on VEP | cortical visual impairment, hyperopia | cortical visual impairment, roving eye movement | Intermittent unilateral divergent strabisms, possibly amblyopia | visual impairment | cortical visual impairment | abnormal VEP and ERG | abnormal VEP and ERG | abnormal VEP and ERG | esotropia, hyperopia, astigmatism | NR | myopia, optic atrophy |
| Cerebellar atrophy or hypoplasia | no | yes, mild | yes, progressive | no | yes, mild | no | yes, severe | yes | yes | yes | yes | yes | NR | yes |
| Other brain MRI findings |  | normal at 7 mo; mild atrophy of the superior vermis and symmetrical atrophy of the superior aspect of the cerebellar hemispheres at 6 yr | thin corpus callosum (CC) | bilateral foci of increased white matter signal- subcortical, periventricular and along cerebellum; cerebral atrophy, thin splenium of CC | no |  | mild diffuse atrophy in supratentorial compartment | cerebral atrophy, CC atrophy, small hippocampus | cerebral  atrophy, thin and foreshortened CC | cerebral  atrophy, thin and foreshortened CC | cerebral  atrophy, thin and foreshortenedCC | foreshortened CC | NA | mild cerebral atrophy, foreshortened CC |
| Other clinical findings |  | feeding difficulties (5 mo); severe sleep apnoea at 3 yr; recurrent chest infections at 7 yr; decreased muscle bulk, high palate | feeding difficulties; hip dysplasia; pectuscarinatum | symmetric growth delay; neurogenic bladder and bowel; OSA and hypoventilation; contrac ture of right hand | hip dysplasia sialorrhea, behavioral difficulties (biting self, screaming) | autism spectrum disorder, plagiocephaly, bifid uvula |  |  |  |  |  |  |  |  |

Abbreviations: DTR – deep tendon reflexes, EEG – electroencephalogram, ERG – electroretinogram, NR – no record, OSA – obstructive sleep apnea, VEP – visual evoked potential, CC – corpus callosum.
