# Supplementary material for: Novel compound heterozygous variants in EMC1 associated with global developmental delay: a lesson from a non-silent synonymous exonic mutation
Source: Front Mol Neurosci. 2023 Apr 28;16:1153156. doi: 10.3389/fnmol.2023.1153156 (PMC10175691; doi:10.3389/fnmol.2023.1153156)
Supplement: Supplementary file 2 [file Presentation_1.PPTX]

## Slide 1
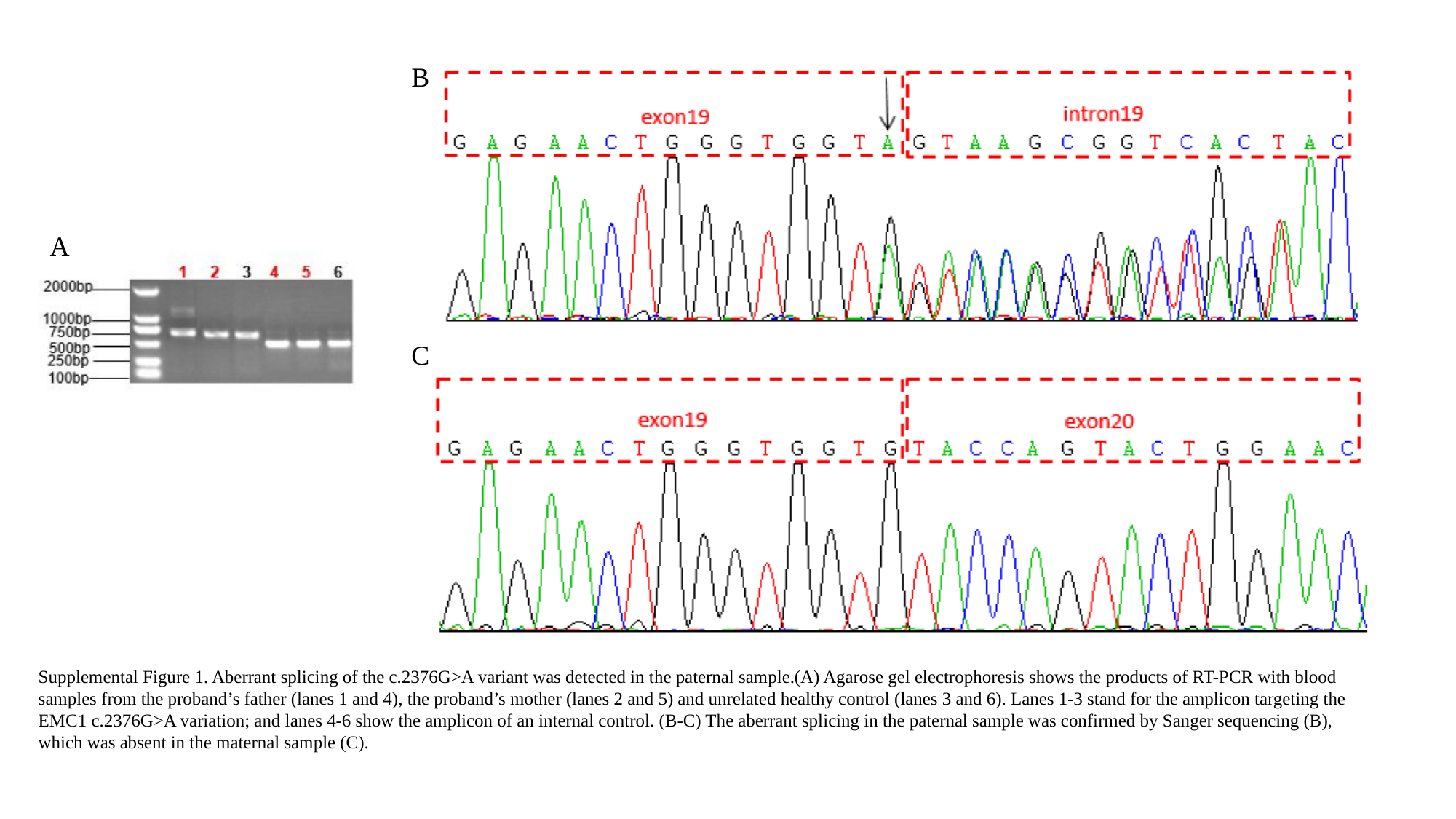

B
A
C
Supplemental Figure 1. Aberrant splicing of the c.2376G>A variant was detected in the paternal sample.(A) Agarose gel electrophoresis shows the products of RT-PCR with blood samples from the proband’s father (lanes 1 and 4), the proband’s mother (lanes 2 and 5) and unrelated healthy control (lanes 3 and 6). Lanes 1-3 stand for the amplicon targeting the EMC1 c.2376G>A variation; and lanes 4-6 show the amplicon of an internal control. (B-C) The aberrant splicing in the paternal sample was confirmed by Sanger sequencing (B), which was absent in the maternal sample (C).
